# The Influence of Topinambur and Inulin Preventive Supplementation on Microbiota, Anxious Behavior, Cognitive Functions and Neurogenesis in Mice Exposed to the Chronic Unpredictable Mild Stress

**DOI:** 10.3390/nu15092041

**Published:** 2023-04-23

**Authors:** Joanna Szala-Rycaj, Aleksandra Szewczyk, Mirosław Zagaja, Agnieszka Kaczmarczyk-Ziemba, Maciej Maj, Marta Andres-Mach

**Affiliations:** 1 Department of Experimental Pharmacology, Institute of Rural Health, Jaczewskiego 2, 20-090 Lublin, Poland; 2 Department of Evolutionary Genetics and Biosystematics, Faculty of Biology, University of Gdansk, WitaStwosza 59, 80-308 Gdansk, Poland; 3 Department of Biopharmacy, Medical University of Lublin, Chodzki 4A, 20-093 Lublin, Poland

**Keywords:** chronic stress, topinambur, inulin, prebiotics, fluoxetine, gut microbiota, neurogenesis, cognitive functions

## Abstract

Daily living and functioning under stress can lead to mental health problems such as anxiety or depression. Over the past decades, a number of studies have been conducted to determine the relationship between the central nervous system (CNS), intestinal flora and bidirectional communication along the gut brain axis (GBA) in the maintaining of homeostasis. One of the most important factors regulating GBA functioning in exposure to stress may be a proper diet enriched in the supplementation with pre-, pro-and synbiotics. In the present study, we examined whether a 10-week oral preventive supplementation with natural prebiotics: topinambur powder (TPB) and chicory root inulin (INU) influenced an anxiety, depressive behavior and cognition in mice exposed to the chronic unpredictable mild stress (CUMS). Additionally, a fluoxetine (FLU) has been used as a reference antidepressive drug. Furthermore, we assessed the effect of TPB, INU and FLU administration on neurogenesis in mice exposed to CUMS and finally analyzed fecal microbiota for possible changes after TPB and INU supplementation in CUMS induced mice. Results obtained from the behavioral studies (elevated plaze maze, forced swim and Morris water maze test) indicated, that 10 week supplementation with TPB (250 mg/kg) and INU (66 mg/kg), similarly to FLU (12 mg/kg), significantly mitigated an anxiety and stress as well as protected learning and memory functions in the CUMS induced mice compared to the control stressed group. Additionally, TPB and INU CUMS mice showed significantly higher level of neurogenesis in comparison to control CUMS group. Interestingly, results obtained from the fecal microbiota analysis showed a beneficial effect of TPB and INU supplementation against CUMS-induced intestinal dysbiosis in mice. In conclusion, the obtained results showed that a long-term, preventive supplementation with TPB or INU alleviates the negative effects such as anxiety, cognitive disorders or dysbiosis in mice exposed to chronic unpredictable stress.

## 1. Introduction

Stress is the body’s natural defensive response to various stimuli and experiences that cause fear, anxiety, or insecurity. The body is able to cope with an acute but short-term stress unlike with chronic stress, which can negatively affect mental health including learning and memory functions [1]. Moreover, stress affects neurogenesis, leading to a decrease in the rate of cell proliferation in the adult hippocampus [2]. In turn, an antidepressant treatment increases hippocampal neurogenesis and may thus block or reverse stress damage [3,4,5].

One of the most important regulators of proper homeostasis is a gut-brain axis (GBA), which connects the central nervous system (CNS), the autonomic nervous system, the enteric nervous system, and the hypothalamic-pituitary-adrenal (HPA) axis [6]. The HPA axis is considered the main efferent axis of stress, and it plays a key role in mental disorders [7]. Stress and elevated pro-inflammatory cytokines activate the HPA axis by secreting corticotrophin, which in turn stimulates the secretion of adrenocorticotropic hormone and finally leads to the release of the main stress hormone cortisol [6]. Stress-induced secretion of glucocorticoids may intensify dysbiosis, i.e., an imbalance of the intestinal barrier, which allows the migration of bacteria with a pro-inflammatory component [8]. Recent clinical studies have shown that intestinal dysbiosis is correlated with the occurrence of depression [9,10]. One of the most significant factors determining the composition and diversity of the intestinal microbiota is diet [11]. An appropriate food selection is very important for the composition and functioning of the intestinal microbiota [12] and affects the diversity of microorganisms [13]. Some bacterial populations produce neurochemicals from food substrates that affect brain and consequently the function of the whole organism [14]. Considering, that a properly balanced diet is one of the most important GBA modifiers, supplementation with pre-, pro- and synbiotics may be a new way to deal with a daily exposure to stress.

The International Scientific Associations of Probiotics and Prebiotics have defined prebiotic as “a selectively fermented ingredient that produces specific changes in the composition and/or activity of the gut microbiota, conferring a health benefit(s) on the host” [15]. The degradation products of prebiotics are mainly short-chain fatty acids (SCFAs), which penetrate the intestinal enterocytes and enter the blood stream affecting all organs, not just the digestive system [16]. Studies by Schmidt et al. [17] showed that prebiotics affect the brain and regulate anxiety and emotional processes. Moreover, they are important in pathogenesis affective disorders, neurodevelopmental diseases and neurodegenerative diseases (e.g., Parkinson’s disease) [18,19,20]. One of the most commonly used natural prebiotics is inulin, a linear fructosyl polymer linked by β-(2,1) bonds (*n* = 3–65), attached to a terminal glucosyl residue by an α-(1,2) bond [21]. Currently, the main sources of inulin used by the industry are chicory and topinambur [22]. Topinamburtubers except inulin and other sugars: starch, fructose, sucrose, maltose consist of water (75–79%), proteins (2–3%), fat (2–3%), some micronutrients (phosphorus, potassium, calcium, magnesium and iron), vitamins C and A and exogenous amino acids, e.g., threonine and tryptophan [23]. The percentage of individual components, including secondary metabolites, depends on many factors, such as a climate and growing conditions, harvest maturity, post-harvest storage time and extraction procedure [24]. TBP due to the high content of inulin and many other nutritional benefits has become an interesting subject of the research mainly in the field of intestinal dysbiosis.

The current study was to examine whether a 10 week preventive oral administration of natural prebiotics: powdered concentrated juice of topinambur (TPB) and powdered inulin from chicory root (INU) affected behavior—in particular anxiety, depression-like behavior, cognition and microbiota in mice exposed to CUMS. Additionally, we performed an analysis of the potential changes in the process of neurogenesis after TPB and INU supplementation in CUMS-induced animals.

## 2. Materials and Methods

### 2.1. Animals and Experimental Conditions

All experiments were performed on 6 week old male C57BL/6J mice (20–25 g). The animals were housed under standard laboratory conditions (natural light-dark cycle, 55 ± 5% humidity and 21 ± 1 °C) with food and water ad libitum. After the 7-day acclimatization period, the animals were randomly assigned to five experimental groups. Each group consisted of seven mice. All experimental procedures were approved by the Local Ethics Committee of the University of Life Sciences in Lublin (No. 73/2020). All study groups were individually housed in a separate room and subjected to chronic unpredictable mild stress (CUMS). The control healthy mice were kept under standard conditions without stress.

### 2.2. Chronic Unpredictable Mild Stress (CUMS)

The CUMS was performed according to the protocols previously described [25,26,27] with minor modifications. In brief, during the 42 days of CUMS-induction, the mice were exposed to 7 different mild stressors, which we has been changed daily so that the type of stressor was unpredictable for the animals. The list of stressors and the time of th exposure to stress stimulus is presented in Table 1.

### 2.3. Drugs

The following substances and drugs were used in the presented study: TPB (purchased form Organic SP.z o.o., Sieniawa, Poland), INU (purchased from FORMEDS Poznań, Poland), FLU (Sigma Aldrich, St. Louis, MO, USA), 5-bromo-2′-deoxyuridine BrdU (Sigma Aldrich Louis, St. Louis, MO, USA), medetomidine hydrochloride (Tocris Bioscience, Bristol, UK), isoflurane (Baxter, Warszawa, Poland). TPB, INU and FLU were suspended in 10 mL water for injection (Baxter, Poland) and orally administered via 1 mL gastric tubes in a volume of 10 mL/kg body weight. The remaining substances except isoflurane (inhalation) were also suspended in 10 mL water for injection and administered intraperitoneally (i.p.) with 1 mL syringes as a single injection, in a volume of 10 mL/kg body weight.

### 2.4. Experimental Study Design

In order to assess the preventive effect of prebiotics on the chronic stress, the mice were divided into 4 groups (*n* = 7): TPB CUMS; INU CUMS; FLU CUMS and Control CUMS (orally administrated with water for injections) before prebiotic supplementation.

To verify a proper induction of the CUMS model, an additional control healthy (Control H, *n* = 7) group of mice receiving water for injections was used in all behavioral test: elevated plaze maze (EPM), forced swimm test (FST) and Morris Water Maze test (MWM).

**Figure 1:**
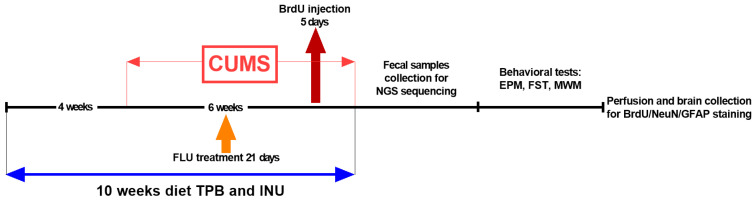
Schematic illustration of the experimental design. TPB-topinambur; INU-inulin; FLU-fluoxetine; BrDU-5-bromo-2′-deoxyuridine; EPM-elevated plus maze test; FST-forced swim test; MWM-Morris water maze test; NeuN-neuronal nuclear cell; GFAP-glial fibrillary acidic protein.

A detailed schematic illustration of the experiments is shown in Figure 1. Briefly, a supplementation with freshly prepared prebiotics (TPB, INU) was initiated 4 weeks prior to CUMS induction and continued for 6 weeks during the exposure to stressors. At the same time Control CUMS animals received water for injection. FLU, as a reference drug, was orally administrated at dose of 12 mg/kg for the last 3 weeks of CUMS induction. The doses of TPB (250 mg/kg) and INU (66 mg/kg) were selected in accordance with the manufacturer’s recommendation. The weight of the animals was monitored three times during the entire experiment (before the start of the experiment, 5th and 10th week of the study). In addition, in the 5th week of the CUMS induction, all animals received a cell proliferation marker BrDU (50 mg/kg) as i.p. injection once daily for 5 days. 10 weeks after the diet, the feces from the rectum were collected for the microbiota analysis, the behavioral tests were performed and then the animals were perfused in order to harvest brains for quantitative analysis of neurogenesis.

### 2.5. Fecal Microbiota Analysis (DNA Extraction, NGS Sequencing, Metagenomic Sequencing)

Genomic DNA samples were extracted from about 15 mg fecal samples using the GeneMATRIX Stool DNA Purification Kit (EurX, Gdańsk, Poland) and following the manufacturer’s protocol. The V3-V4 hypervariable regions of the bacterial 16S rRNA gene were amplified using primers 341F/785R 29 [28]. Details were congruent with methodology described in Kaczmarczyk-Ziemba et al. [29] and Szewczyk et al. [30]. Raw NGS data are deposited and fully available in the Sequence Read Archive (accession number PRJNA914996).

### 2.6. Behavioral Tests

10 weeks after the diet, the behavioral tests evaluating an anxiety and depressive symptoms as well as learning and memory functions were performed. To verify the proper induction of the CUMS model, in addition to Control CUMS group, we used a Control H mice in all the behavioral tests (*n* = 7).

#### 2.6.1. Elevated plus Maze Test, EPM

The EPM test is based on the natural aversion of rodents to open spaces and can be used to assess anxiety-related behavior in rodent models. The EPM consisted of two open arms (50 cm × 10 cm) and two closed arms (50 cm × 10 cm, surrounded by 40 cm plastic walls) that extended from a common central platform (10 cm × 10 cm). During the behavioral test, the mouse was individually placed in the central area, with the head pointing towards the closed arm, and allowed to explore the maze for 5 min [31]. The proportion of time spent in open arms (time in open arms/5 min) was assessed and calculated using the VideoMot2 video tracking system (TSE Systems, Berlin, Germany). The device was cleaned with an ethanol solution (70%) and dried before and after each test session to remove odors from the entire device that could interfere with the task.

#### 2.6.2. Forced Swim Test, FST

FST (known as the despair behavior test) is a swim test enabling an analysis of thedepressive behavior in rodents. Animal’s behavior was assessed according to the Detke method [32] during a 5-min proper test and the immobility time was analysed [33,34]. Data were analyzed using VideoMot2 video tracking system (TSE System, Berlin, Germany).

#### 2.6.3. Morris Water Maze Test, MWM

An assessment of potential cognitive impairments was done using the MWM test according to the methods described in our previous studies [30,35]. The mouse swimming patterns were recorded using the VideoMot2 video tracking system (TSE Systems, Berlin, Germany). We performed one daily session of four 60-s trials (each trial representing a different pool quadrant) for 5 consecutive days. Twenty-four hours after the last training, the final test (probe test) was performed. Three parameters were measured: escape latency, distance and time spent in the W channel.

### 2.7. Immunohistochemistry Staining

#### BrdU/NeuN/GFAP Staining

**Figure 2:**
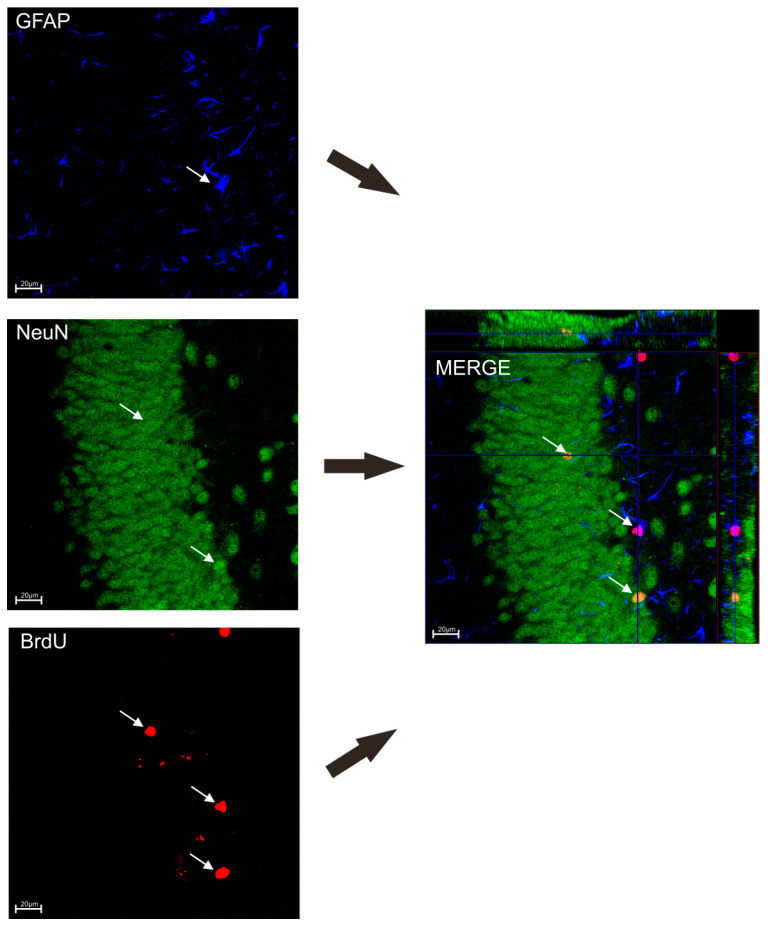
Co-localization of BrdU+ cells with neurons (NeuN) and astrocytes (GFAP). The white arrow points to GFAP stained blue, NeuN stained green, BrdU+ cells stained red.

To determine the influence of TPB and INU diet on neuronal survival, mice were transcardially perfused 3 weeks after the last injection of BrDU according with the procedures described in our previous studies [35,36,37]. The brains were removed, processed, and coronal sections were cut on a 50 μm thick vibratome. The free-floating section was immunostained with the procedures described in our previous studies [35,37,38]. In order to estimate the number of BrdU-positive (BrdU+) cells in the subgranular zone (SGZ), at least 12 one-in-six sections were scored per animal. All counts were restricted to the dentate granule cell layer (GCL) and a 50 μm boundary along the hilar margin that includes the SGZ. The total number of BrdU+ cells exhibiting neuron-specific (NeuN) or astrocyte-specific (GFAP) markers was determined using confocal microscopy to assess BrdU co-localization and phenotypic indices in representative sections from each animal (Figure 2). Confocal microscopy was performed using the Nikon A1R confocal microscope in cooperation with the Department of Biochemistry and Molecular Biology of the Medical University of Lublin.

### 2.8. Statistical Analysis of the Results

Data (EPM, FST, MWM tests and neurogenesis) were analyzed by one-way ANOVA using commercially available GraphPad Prism version 8.0 for Windows (GraphPad Software, San Diego, CA, USA). Dunnett’s test was then used for multiple comparisons. All data were expressed as means with their standard errors (S.E.M).

The following analyzes were used to determine the composition of the microbiome:

- Alpha diversity measures using Chao1 and Shannon indices were calculated using the Microbiome Analyst platform. The non-parametric Kruskal-Wallis test was used to compare differences in alpha diversity between different groups.
- Principal Coordinate Analysis (PCoA) was used to show species diversity between samples using Primer 7 software.
- Primer 7 was used for similarity analysis (ANOSIM) and multivariate analysis of variance based on permutations (PERMANOWA).
- The Past 4.0 program was used to perform the SIMPER analysis.
- LEfSe analysis was performed using the Microbiome Analyst platform.

## 3. Results

### 3.1. The Impact of TPB, INU and FLU on the Body Weight of Mice Exposed to CUMS

**Figure 3:**
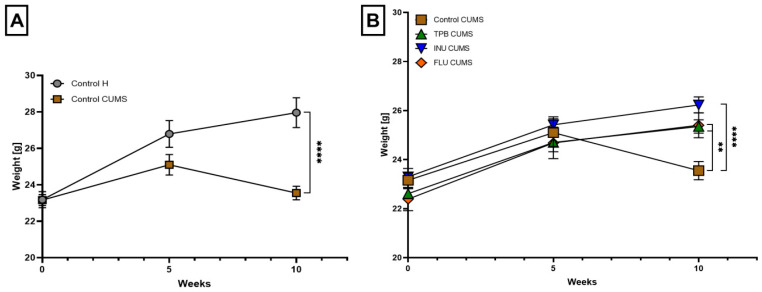
The impact of TPB, INU and FLU on the body weight of mice exposed to CUMS. The influence of the CUMS induction on the weight gain in mice (**A**). The effect of CUMS induction on the body weight of the mice administrated with TPB, INU and FLU (**B**). The results were analyzed by *t*-student’s test (**A**) and one-way analysis of variance (ANOVA) followed by Dunnett’s test for multiple comparisons (**B**). Each bar represents the mean for seven mice; error bars are S.E.M. (** *p* < 0.01, **** *p* < 0.0001; *n* = 7).

The analysis of weight gain in mice showed a statistically significant decrease in the body weight of the Control CUMS mice compared to the Control H group (23.55 ± 0.37 vs. 27.96 ± 0.82, respectively, *p* < 0.0001; *n* = 7, Figure 3A). During the induction of the CUMS model, the INU-treated mice showed the highest tendency to gain weight compared to the CUMS control group (26.23 ± 0.33 vs. 23.55 ± 0.37, *p* < 0.0001; *n* = 7, Figure 3B). Mice receiving TPB and FLU also gained more weight than the Control CUMS group (25.35 ± 0.27, 25.40 ± 0.51 vs. 23.55 ± 0.37, respectively, *p* < 0.01; *n* = 7, Figure 3B.)

### 3.2. The Influence of a Prebiotic Diet and FLU on the Depressive Behavior of Mice Exposed to CUMS

**Figure 4:**
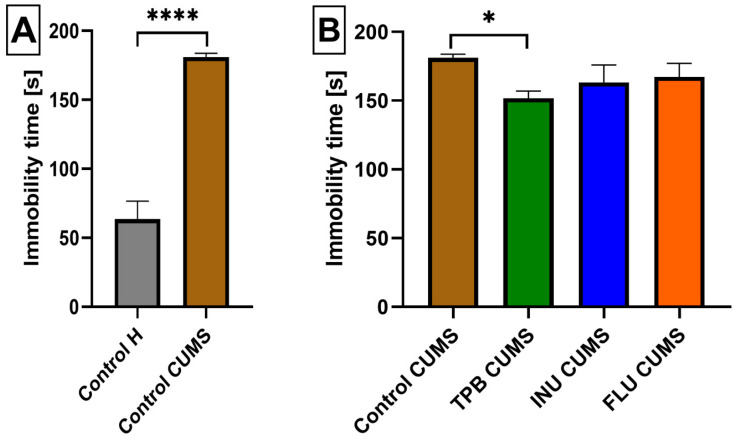
The impact of a prebiotic diet and FLU on the depressive behavior of mice exposed to CUMS. The influence of CUMS induction on the immobility time inmice (**A**). The effect of TPB, INU and FLU administration on anxiety-like behavior in the FST test after CUMS induction in mice (**B**). The results were analyzed bywere analyzed by *t*-student’s test (**A**) andone-way analysis of variance (ANOVA) followed by Dunnett’s test for multiple comparisons (**B**). Each bar represents the average of seven mice; error bars are S.E.M. (* *p* < 0.05, **** *p* < 0.0001; *n* = 7).

To verify a proper CUMS induction, we analyzed the FST data of Control CUMS and Control H mice and we observed a statistically significant increase in the immobility of Control CUMS mice compared to the Control H group (181.1 ± 2.650; 63.5714 ± 12.98, *p* < 0.0001, *n* = 7; Figure 4A).

Analyzing subsequent FST results for all CUMS study groups, we noticed, that TPB mice had a significantly lower immobility time (151.7 ± 5.268, *p* < 0.05, *n* = 7, Figure 4B) than the Control CUMS group (181 ± 2.650; *p* < 0.05, *n* = 7). Similarly, INU and FLU CUMS mice also indicated a slightly decreased immobiliy time, however not statistically significant with respect to the Control CUMS group (Figure 3B).

### 3.3. The Impact of a Prebiotic Diet and FLU on Anxiety Behavior of Mice Exposed to CUMS

**Figure 5:**
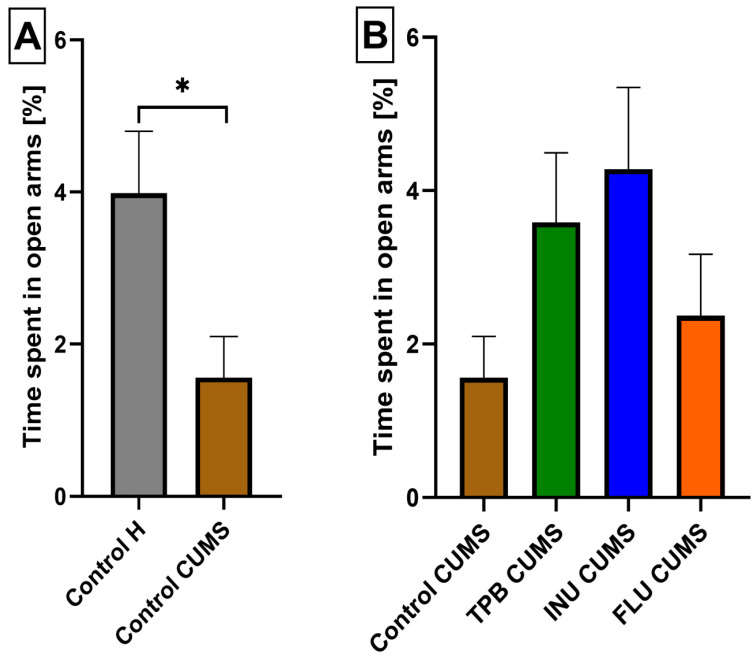
The impact of a prebiotic diet and FLU on anxiety behavior of mice exposed to CUMS The influence of CUMS induction on the anxiety behavior in mice (**A**). The impact of the TPB, INU and FLU administration on the anxiety-like behavior in EPM test after CUMS induction in mice (**B**). The results were analyzed using *t*-student’s test (**A**) and one-way analysis of variance (ANOVA), followed by Dunnett’s test for multiple comparisons (**B**). Each bar represents the mean for seven mice; error bars are S.E.M. (* *p* < 0.05; *n* = 7).

**Figure 6:**
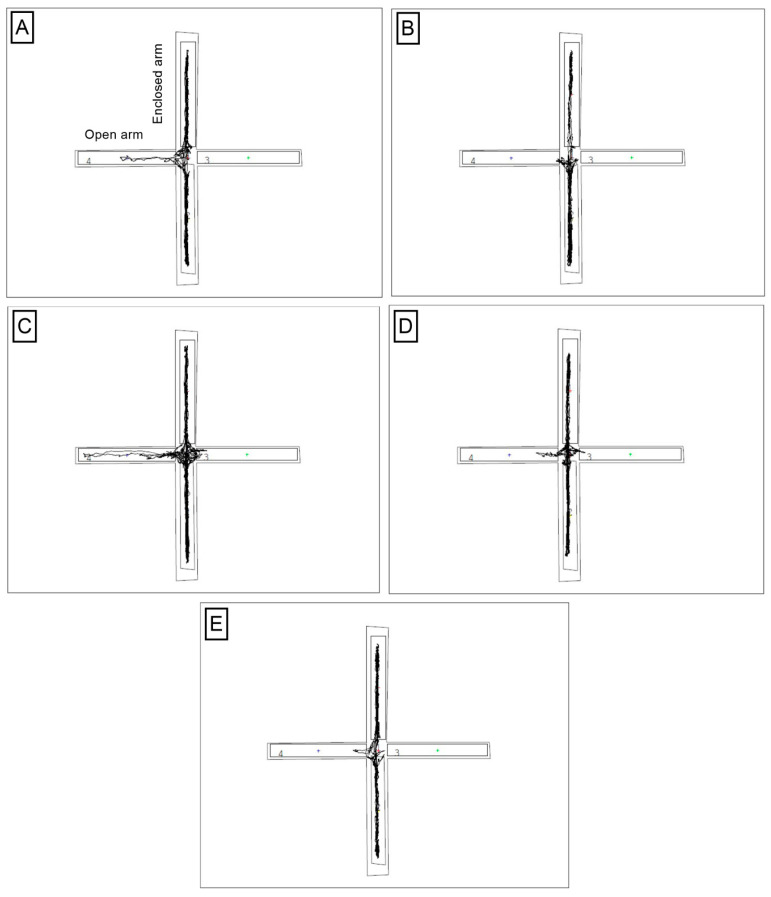
Sample recordings of the exit path to open arms for one selected animal from each study group. (**A**)-Control H; (**B**)-Control CUMS; (**C**)-INU CUMS; (**D**)-TPB CUMS; (**E**)-FLU CUMS. The recordings were made with a camera and a computer system with the Video Mot2 System software by TSE System.

To verify the anxiety behaviors in the Control CUMS mice, the EPM test was performed and the obtained data were compared to the Control H mice indicating a statistically significant decrease of time spent in the open arms for the CUMS group (1.560 ± 0.5389, 3.986 ± 0.8128, respectively, *p* < 0.05, *n* = 7, Figure 5A and Figure 6A,B). Likwise, the analysis of the results from TPB, INU and FLU administration in CUMS-induced mice indicated a higher percentage of time spent in the open arms, however, without statistical significance (Figure 5B and Figure 6B–E).

### 3.4. The Effects of a Prebiotic Diet and FLU on Spatial Learning and Memory of Mice Exposed to CUMS

**Figure 7:**
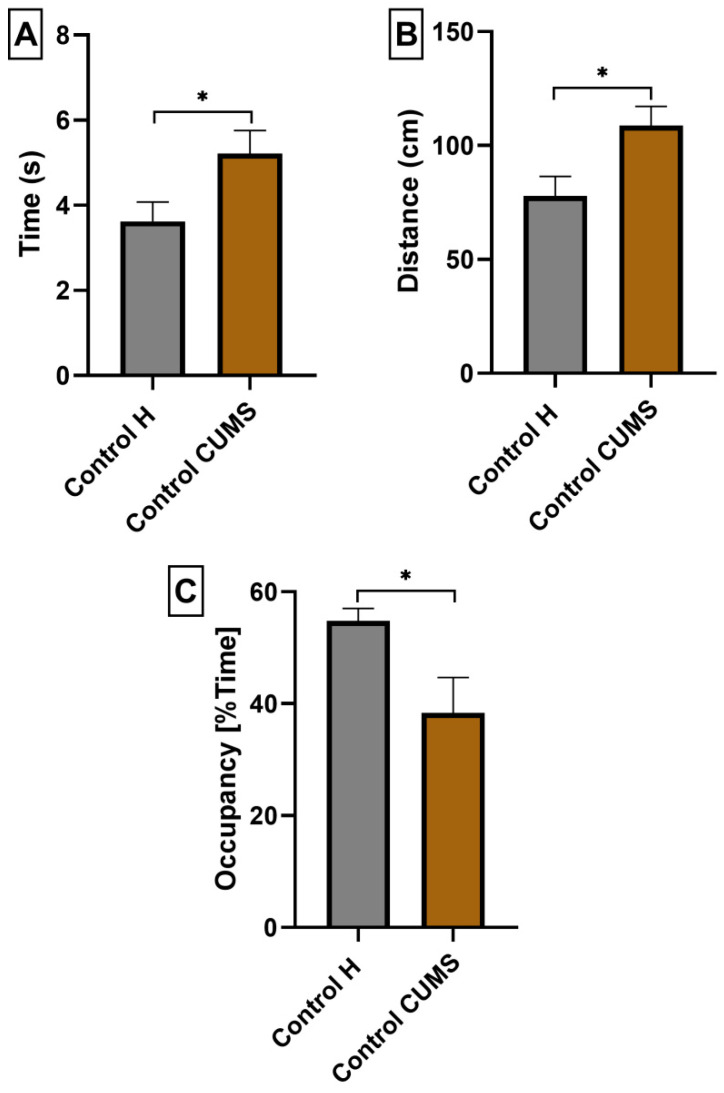
The influence of CUMS induction on escape latency (**A**), total distance (**B**) and mean percentage of time spent in the W channel (**C**) of mice in the MWM test. The results were analyzed using *t*-student’s test. Each bar represents the mean for seven mice; error bars are S.E.M. (* *p* < 0.05; *n* = 7).

To confirm cognitive impairment in CUMS induced mice, we performed the MWM test and compared the obtained data with the results of Control H mice. Three selected parameters were analyzed: (A) the average time needed to find the platform from all quadrants, (B) the average distance traveled in order to find the platform from all quadrants, and (C) the mean percent of time spent in the W-Channel from all quadrants. The mean escape latency (5.220 ± 0.5391; *p* < 0.05; *n* = 7; Figure 7A),the mean distance (103.8 ± 8.406; *p* < 0.05; *n* = 7; Figure 7B) and the mean percentage of time spent in the W channel averaged over the four quadrants (38.33 ± 6.353; *p* < 0.05; *n* = 7; Figure 7B) were significantly worse for Control CUMS mice compared to Control H mice (3.624 ± 0.4547, 77.78 ± 8.685, 54.76 ± 2.248, respectively; *n* = 7, Figure 7A–C).

In order to identify possible changes in the cognitive functions after the TPB, INU and FLU administration in mice exposed to CUMS, the animals were subjected to the MWM test and all study groups indicated significantly better results compared to the Control CUMS mice.

**Figure 8:**
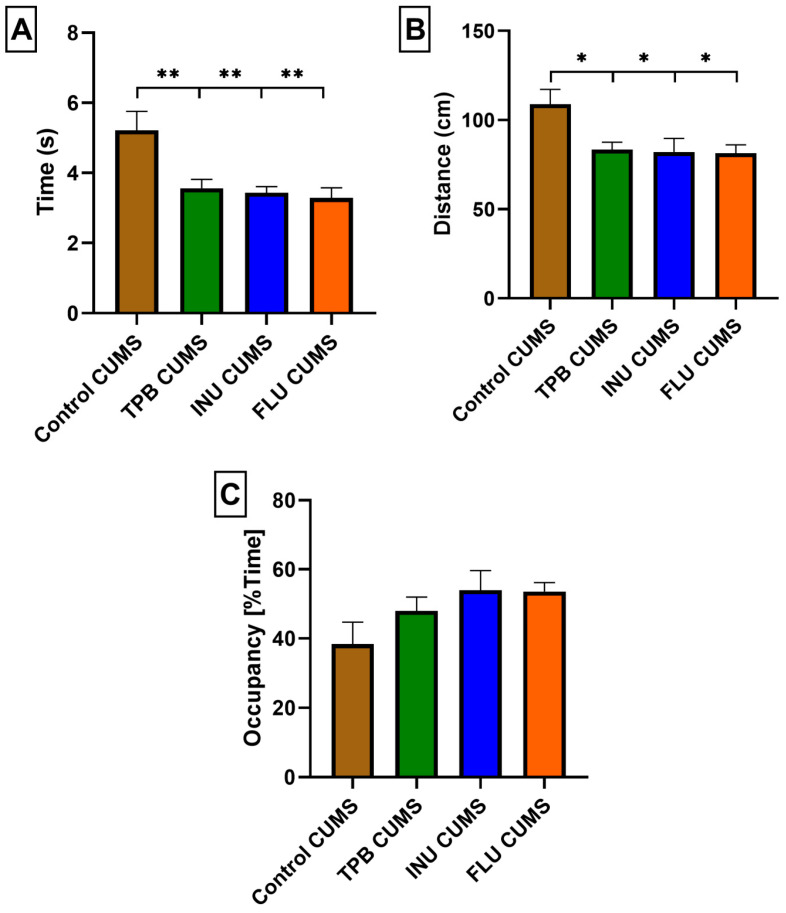
Evaluation of the effect of a long term TBP, INU and FLU administration on escape delay (**A**), total distance (**B**), and mean percent of time spent in the W-channel (**C**) in mice after CUMS induction in the MWM test. The results were analyzed using one-way analysis of variance (ANOVA), followed by Dunnett’s test for multiple comparisons. Each bar represents the mean for seven mice; error bars are S.E.M. * *p* < 0.05 and ** *p* < 0.01; *n* = 7).

The mean escape latency for TPB CUMS mice (3.565 ± 0.2525; *p* < 0.01; *n* = 7; Figure 8A) as well as the mean distance (83.48 ± 4.037; *p* < 0.05; *n* = 7; Figure 8B) were significantly more favorable compared to the Control CUMS mice (5.220 ± 0.5391, 108.8 ± 8.406, respectively; *n* = 7, Figure 8A,B).

Similarly, INU CUMS and FLU CUMS mice showed significantly better mean escape latency (3.436 ± 0.1713, 3.291 ± 0.2876, respectively; *p* < 0.01; *n* = 7; Figure 8A) and mean distance (82.05 ± 7.626, 81.46 ± 4.613, respectively; *p* < 0.05; *n* = 7; Figure 8B) in comparison with Control CUMS group.

The mean percentage of time spent in the W channel averaged over the four quadrants and was more favorable for TPB, INU and FLU CUMS mice, although the differences were not statistically significant compared to the Control CUMS group (Figure 7C).

### 3.5. Effect of TPB, INU and FLU Administration on Neurogenesis in the SGZ and the GCL of theMouse Hippocampus Exposed to CUMS

**Figure 9:**
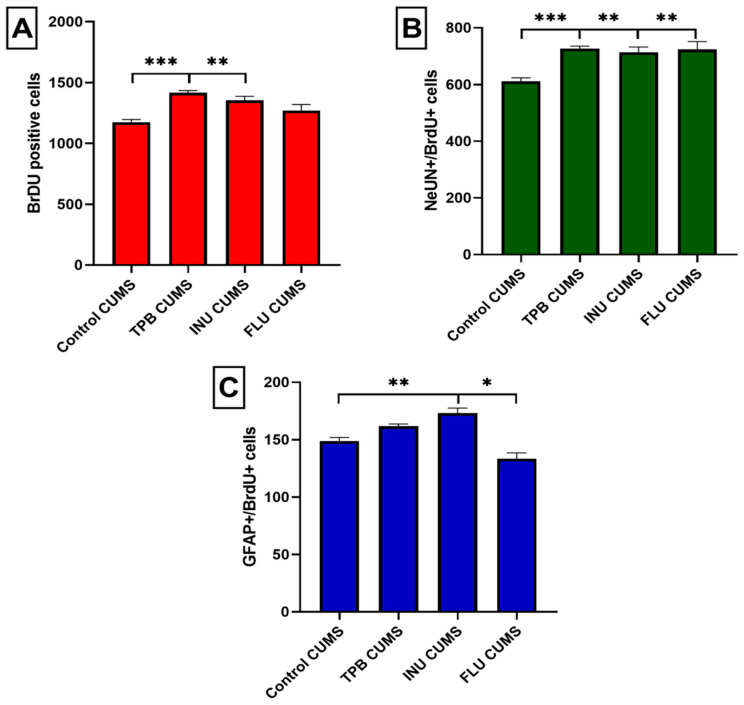
Quantitative analysis of the process of neurogenesis. Effect of TPB, INU and FLU on the total number of newborn cells (**A**), newborn neurons (**B**) and newborn astrocytes (**C**) in the dentate SGZ in mice after CUMS induction. Cell numbers represent an estimate of the total number of positively labeled cells in the SGZ in both hemispheres. The results were analyzed by one-way analysis of variance (ANOVA) followed by Dunnett’s test for multiple comparisons. Each bar represents the average of five mice; error bars are S.E.M. (* *p* < 0.05, ** *p* < 0.01 and *** *p* < 0.001; *n* = 5).

Quantitative analysis of potential changes in the process of neurogenesis was performed for all study CUMS groups in relation to the Control CUMS mice as a reference group. Obtained results revealed a clear difference between Control CUMS mice and TPB CUMS mice in the total number of BrDU positive cells (1174 ± 23.13 vs. 1417 ± 17.12, respectively, *p* < 0.001; *n* = 5; Figure 9A). Similarly, INU CUMS mice showed a significant increased in the total number of BrDU + cells to 1354 ± 33.76 (*p* < 0.01; *n* = 5) compared to the Control CUMS mice. The FLU CUMS group (1271 ± 48.50, *n* = 5, Figure 9A) also had a higher newborn cell count compared to the Control CUMS mice, however, without statistical significance.

In order to assess the differentiation of newly formed cells labeled with the BrdU marker, immunohistochemical staining of the colocalization of BrdU/NeuN and BrdU/GFAP-positive cells was performed. The analysis of the obtained results showed that the number of cells labeled with BrdU/NeuN was significantly higher in mice receiving TPB when compared to the Control CUMS group (727 ± 8.781 vs. 611.4 ± 11.93, respectively *p* < 0.001; *n* = 5; Figure 9B). Likewise, the mean number of BrDU/NeuN+ cells significantly increased for INU (714.2 ± 17.90; *p* < 0.01; *n* = 5; Figure 9B) and FLU mice (724 ± 27.68; *p* < 0.01; *n* = 5; Figure 9B) compared to the Control CUMS group.

Quantitative analysis of the BrDU/GFAP+ cells colocalization indicated lack of statistically significant differences for TPB CUMS mice in the level of astrocytes compared to the CUMS Control group (161.8 ± 1.934 vs. 149 ± 3, *n* = 5; Figure 9C). In turn, a clear increase of BrDU/GFAP+ cells was observed for the INU group (173 ± 4.341; *p* < 0.01; *n* = 5) compared to the Control CUMS mice. Interestingly, a statistically significant reduction in the number of BrDU/GFAP+ cells was observed in the FLU group (133.4 ± 5.163; *p* < 0.05; *n* = 5) compared to the CUMS control group.

### 3.6. Effect of TPB, INU and FLU on the Composition of the Intestinal Microbiota in Mice Exposed to CUMS

**Figure 10:**
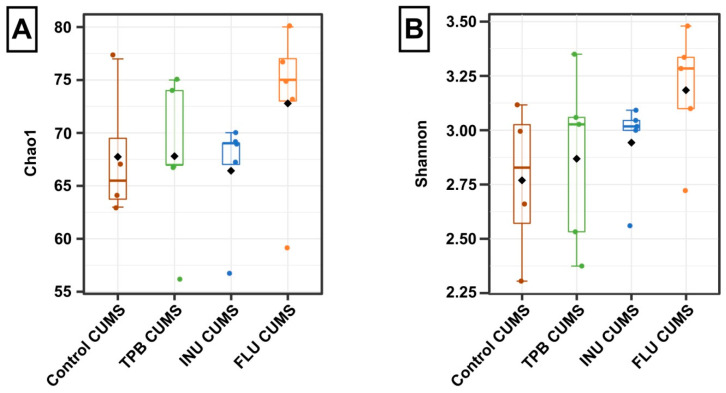
Alpha diversity indices calculated for groups: Control CUMS, TPB CUMS, INU CUMS and FLU CUMS. (**A**)—Chao1 index; (**B**)—Shannon index. Boxes represent the interquartile range (IQR) and the median. Whiskers represent 1.5× the IQR. Diamonds represent the mean. The results were analyzed using the Kruskal-Wallis test. Each bar represents the mean for 5 mice. Data are presented as the mean ± S.E.M.

An analysis of potential changes in the composition of the intestinal microbiota was performed for all study CUMS groups in relation to the Control CUMS mice as a reference group. Alpha diversity analysis revealed subtle differences in the microbial community composition of each group. The value of the Chao1 index was the highest in the FLU CUMS group (Figure 10A), however, with no significant differences between the Chao1 indices (H = 2.8461, *p* = 0.42). A similar pattern was observed for the Shannon index, where the highest value was observed in the FLU CUMS group (Figure 10B) but without significant differences (H = 4.5095, *p* = 0.21).

**Figure 11:**
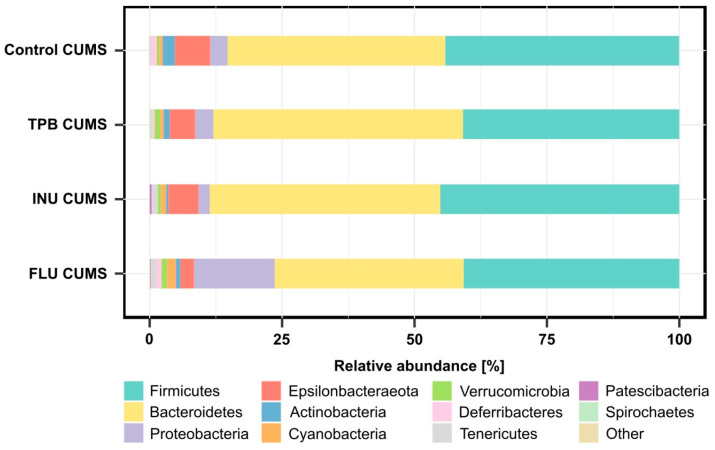
Relative abundance of bacterial phyla identified in tested groups of samples. Each bar represents the mean for 5 mice.

Among the analyzed microbial communities (Supplementary Table S1), 11 types of bacteria were specified. Firmicutes and Bacteroidetes were the most abundant in all tested samples (Figure 11). Moreover, the abundance of Proteobacteria was the highest in the FLU CUMS group (15.2%) compared to the Control CUMS, INU CUMS and TPB CUMS, where the abundance was lower than 3.5%. In addition, all groups had reduced numbers of Epsilonbacteraeota (Control CUMS-6.6%, INU CUMS-5.7%, TPB CUMS-4.8%, FLU CUMS-2.6%) and Actinobacteria (Control CUMS-2.4%, INU CUMS-0.4%, TPB CUMS-1.2%, FLU CUMS-0.9%).

**Figure 12:**
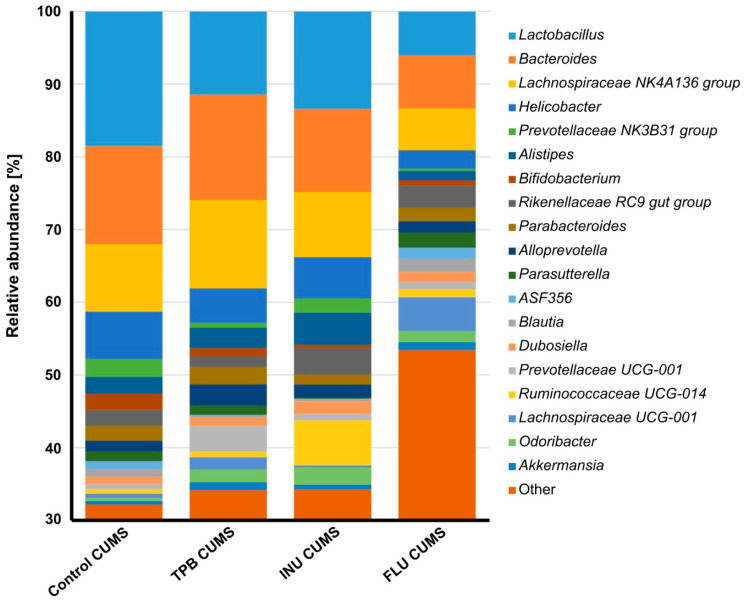
Relative abundance of bacterial genera identified in at least two tested groups of samples with the abundance above 1%. Each bar represents the mean for five mice.

At the genus level, 19 genera were identified with abundances higher than 1% in at least two study groups (Figure 12, Supplementary Table S1). A decreased in relative abundance of Lactobacillus, Bacteroides, Lachnospiraceae NK4A126 and Helicobacter were observed in the FLU CUMS group. In the same group, the number of the following increased: Lachnospiraceae UCG-001 (FLU CUMS-4.6% vs. <1.7% in other study groups), Enterobacter (FLU CUMS-2.4% vs. <1.0% in other study groups) and Escherichia-Shigella (FLU CUMS-3.3% vs. <1.0% in other study groups). The INU CUMS group was distinguished by the more numerous type of Ruminococcaceae UCG-014 compared to the other study groups (6.2% vs. <1.2%). Increased abundance of Prevotellaceae UCG-001 was observed for TPB CUMS compared to the rest of the tested groups (6% vs. <1.1%).

**Figure 13:**
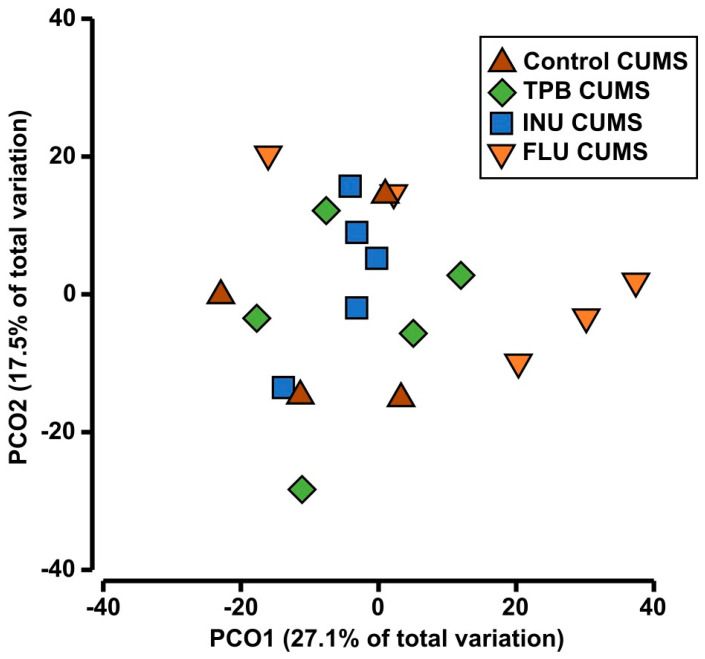
Principal-coordinate analysis (PCoA) showing a two-dimensional ordination of gut microbiota profiles determined for groups: Control CUMS, TPB CUMS, INU CUMS and FLU CUMS. Each point represents one mice from the group.

Results of ANOSIM analysis confirmed lower distances among intragroup samples than among intergroup samples (R = 0.217, *p* = 0.018). The PCoA analysis showed a high similarity between the studied profiles grouped by the treatment (Figure 13). However, PERMANOVA found significant differences between the study groups (Pseudo-F = 1.6949, *p* = 0.016). A further pairing test confirmed significant differences only between the FLU CUMS and INU CUMS groups (t = 1.7105, *p* = 0.015). The overall mean difference between the two groups was estimated at 46.09%. The SIMPER analysis identified thirteen taxa of bacteria associated primarily with the observed differences in FLU CUMS and INU CUMS profiles (i.e., Lactobacillus, Bacteroides, Ruminococcaceae UCG-014, Lachnospiraceae UCG-001, Helicobacter, Lachnospiraceae NK4A136 group, Escherichia-Shigella, Rikenellaceae RC9, Alistipes, Enterobacter, Parasuterella, as well as unidentified members of the Enterobacteriaceae and Muribaculaceae families). In addition, five of these taxa (i.e., Lactobacillus, Bacteroides, Helicobacter, Lachnospiraceae group NK4A136, and unidentified members of the family Muribaculaceae) were found in all pairwise comparisons as significantly shaping differences among tested groups.

**Figure 14:**
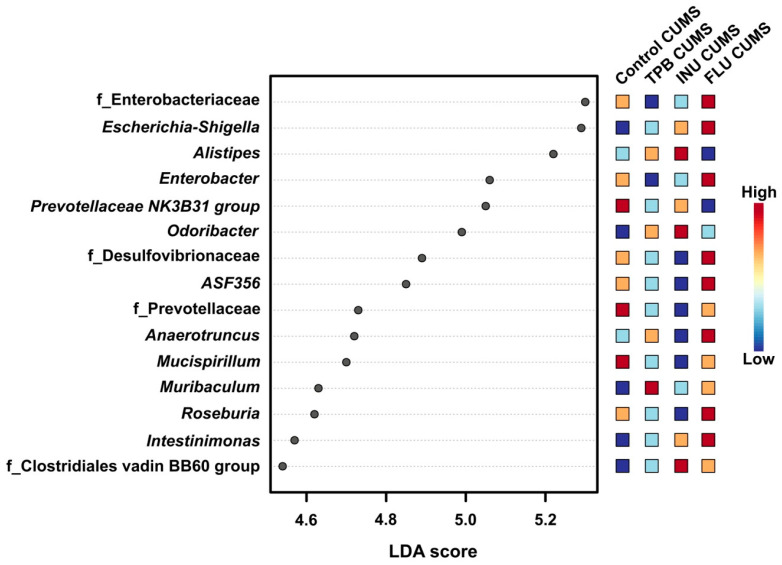
LEfSe analysis of intestinal microflora profiles of the following groups: control CUMS, TPB CUMS, INU CUMS, FLU CUMS. The histogram of LDA results reveals the most numerous taxa among the studied groups.

LEfSe analysis was performed to identify the bacterial taxa responsible for differences in beta diversity (*p* value cutoff = 0.2, log LDA score = 2.0). Thirteen bacterial taxa were found to show differences in the gut microbiota of Control CUMS mice (Figure 14). LEfSe results showed three biomarker taxa for the Control CUMS group (i.e., Prevotellaceae NK3B31, Alistipes, Mucispirillum). Eight biomarker taxa were found for the FLU CUMS group: Enterobacteriaceae, Escherichia-Shigella, Enterobacter, Desulfovibrionaceae, Lachnospiraceae ASF356, Anaerotruncus, Roseburia and Intestinimonas. In turn, for INU-CUMS, three taxa of biomarkers were identified: Alistipes, Odoribacter, Clostridialesvadin BB60 group. Muribaculum was the biomarker for the TPB CUMS group in the LEfSe analysis.

## 4. Discussion

In recent years we can observe a significant increase in scientific research aimed at evaluating the relationship between supplementation with pro- pre- or synbiotics and stress or mental health disorders. Long-term stress may lead to changes in the intestinal microflora [39], causing an increase in pro-inflammatory cytokines [40]. It is well known that inflammation is one of the triggers of neurodegenerative and neuropsychiatric disorders such as depressive disorders [41], schizophrenia [42], Parkinson’s disease [43] and Alzheimer’s disease [44]. Numerous studies suggest that prebiotic supplementation with inulin, galactooligosaccharide, polydextrose, fructooligosaccharide may protect the host from the negative effects of stress [45,46,47,48,49].

While there is a lot of information about prebiotics and their impact on the gut microbiota and stress, it’s hard to find data on plants containing natural prebiotics such as TPB and their impact on stress, neurogenesis and cognitive functions in the exposition to the chronic mild unpredictable stress. Thus, the aim of our study was to evaluate the efficacy of the preventive TPB supplementation on mice exposed to CUMS. Additionally, we tested the potential of a pure prebiotic substance INU isolated from chicory root and FLU as a reference antidepressive drug in the CUMS-induced mice. First, we checked whether a preventive supplementation with TPB and INU, might have a protective effect on anxious (EPM) and depressive (FST) behavior during CUMS induction in mice. The obtained results showed that TPB at a dose of 250 mg/kg significantly reduced the immobility time in the FST test, while INU (66 mg/kg) showed a similar, but slightly lower potential compared to the Control CUMS mice. In turn, the EPM test indicated that INU CUMS group had the longest percentage of time spent in the open arms followed by TPB CUMS compared to the Control CUMS mice. In recent years INU-like oligosaccharides isolated from various plant species have become a very interesting research topic for scientists. Beneficial results for INU-type oligosaccharides isolated from *Smallanthus sonchifolius* were presented by An et al. [50], who assessed their an antidepressant effect in behavioral models of depression in rats. The doses of 25, 50 and 100 mg/kg significantly reduced the immobility time in the tail suspension test and in the FST test. In turn, Qiu et al. [45] obtained inulin-type oligosaccharides from *Morinda officinalis* and evaluated their effect on behavioral deficits in a model of posttraumatic stress in rats. Obtained results from the EPM test indicated that INU (25–100 mg/kg) reversed the reduced time and open arm entries for tested animals compared to the stressed control rats. In turn Guo et al. [51] investigated whether INU could alleviate the symptoms of schizophrenia by modulating the gut microbiota and inhibiting microglia activation through the microbiota-GBA in a mouse model of schizophrenia. The open field test (OFT) used to assess the overall level of anxiety showed that INU at a dose of 2 g/kg alleviated schizophrenia and depressive behavior. Recent research by Cruz-Pereira and colleagues [52] showed that supplementation with FOS-inulin ameliorates the stress response in aged mice at a behavioral and metabolic level with no relation to the immune system or HPA axis but through the regulation spermine and 4-Hydroxybenzaldehyde in the prefrontal cortex. Taking into account the above data from in vivo studies, we can conclude that prebiotic supplementation may play an important protective role against the influence of stress factors.

Surprising data we obtained for the mice receiving a reference antidepressant FLU (12 mg/kg) during the last 3 weeks of CUMS induction. Results from the FST and EPM test indicated only a slight anxiolytic and antidepressive effect of FLU. In turn, Tunc-Ozcan et al. [53] showed less immobility in the FST, more time spent in open arms in the zero maze test and a higher percentage of distance to the center of the ventricle in the OFT, after a 2 weeks FLU i.p. injection (10 mg/kg) in the mouse CUMS model, which suggests a reduction in depression and anxiety behavior. The lower anxiolytic and antidepressant effectiveness of FLU in our studies may be due to the fact, that different routes of the drug administration were used in both experiments and an i.p. administration may induce a better effect.

In the next stage of our research, we assessed the influence of TPB, INU and FLU on cognitive functions in CUMS induced mice. All study groups showed significantly better time and distance to reach the platform compared to the Control CUMS mice. MWM results from our previous studies evaluating the effect of a 10-week diet of TPB (250 mg/kg) and INU (66 mg/kg) on healthy mice showed no statistically significant differences in the time and distance needed to find the platform in comparison to a healthy control mice, whereas FLU (12 mg/kg) significantly disturbed the time and distance parameters [30]. Similar results confirming the beneficial effect of inulin on cognitive functions were presented by Messaoudi et al. [54] investigating the effectiveness of oral administration of oligofructose enriched inulin at doses of 5 and 10% on the behavior and cognition of male Wistar rats. Results they obtained from the light-extinction test indicated that inulin + oligofructose improves cognitive performance in tested rats. In turn, Guo et al. [51] showed that a 6-week inulin supplementation at a dose of 2 g/kg improved learning and spatial memory in a mouse model of schizophrenia.

Our neurogenesis study results indicated strong protective properties of TPB and INU preventive supplementation on the stem cell proliferation compared to Control CUMS mice, whereas FLU administration slightly increased BrDU+ cells in the tested FLU CUMS group. Our previous results concerning TPB and INU supplementation on healthy mice indicated no changes in BrDU+ cells, whereas 2 weeks administration of FLU (12 mg/kg) statistically significantly reduced the number of BrDU+ cells compared to the healthy control group [30]. Similarly, David et al. [55] evaluated the effect of a 3-week treatment with FLU in a model of anxiety and depression caused by over-exposure to glucocorticoids in C57BL/6Ntac mice. Results they obtained indicated that FLU administrated i.p. at a dose of 18 mg/kg completely abolished the corticosterone-induced reduction of neurogenesis. Additionally, FLU treatment had no effect on neurogenesis in corticosterone-naive mice. In turn, Tunc-Ozcan et al. [53] determined the role of neonatal neurogenesis and neuronal activity in the antidepressant effect of FLU in a mouse model of CUMS. They showed that treatment with FLU (10 mg/kg) significantly increased the total number of the dividing cells. Interesting findings from Szewczyk et al. [30] studies using healthy C57BL/6J mice showed that 10-week administration of TPB and INU did not change the BrdU+ cells compared to the control group, while 3 weeks administration of FLU (12 mg/kg) statistically significantly reduced the number of BrDU+ cells compared to the healthy control group. Based on the above studies, it can be concluded that the use of FLU in depressive models may have a positive effect on newborn cell proliferation and neurogenesis, in contrast to the effects of FLU on healthy brains.

In the last stage of our study, the 16S rRNA sequencing was performed to assess the possible changes in the mouse gut microbiome after CUMS induction. The gut microbiota of Control CUMS mice showed the presence of 3 biomarker taxa, i.e., *Prevotellaceae NK3B31*, *Alistipes* and *Mucispirillum*. In addition, bacteria of the *Actinobacteria* genus were dominant in this group. An interesting clinical study concerning the impact of depression on the microbiota was showed by Jiang et al. [56]. They analyzed stool samples from 46 depressed patients and 30 healthy controls. Pyrosequencing showed an increase in *Actinobacteria* and *Alistipes* in people with depression. In turn, Rong et al. [57] studied differences in the gut microbiota of patients with depression and patients with a depressive episode in bipolar disorder. The microbial community in patients with depression was characterized by an increased abundance of Prevotellaceae, including *Prevotelladenticola F0289*, *Prevotella intermedia 17*, *Prevotella ruminicola* and *Prevotella intermedia*. Interesting results from in vivo studies presented Zhang et al. [58], using CUMS-induced depression mouse model. The results from the evaluation of the gut microbiota in Control CUMS mice indicated the following dominant species at the genus level: *Odoribacter*, *Mucispirillum*, *Dehalobacterium*, *Oscillospira*, *Ruminococcus*, *Bilophila*, *Desulfovibrio* and *Helicobacter*.In our study, the most characteristic biomarker was *Muribaculum*, which naturally occur in C57BL/6J mice and participates in the polysaccharides decomposition and thus in maintaining normal conditions in the intestines of mice [59]. Additionally, the TPB CUMS group was distinguished by the most numerous type of *Prevotellaceae UCG-001*. Interestingly, our previous study by Szewczyk et al. [30] on healthy C57BL/6J mice also showed an increase in this type of bacteria in the INU group after 10 weeks of supplementation. *Prevotella* produces succinates that participate in the degradation of inulin [60] and TPB is 80% composed of inulin, which may explain an increased abundance of this bacteria.

Analyzing the microbiome results obtained for the INU CUMS group, we noticed an increased number of *Ruminococcaceae UCG-014*, and its characteristic biomarkers including: *Alistipes*, *Odoribacter* and Clostridielesvadin BB60. *Ruminococcaceae UCG-014* inhabits the colon and cecum and is considered to be one of the SCFAs producers. Likewise, it is responsible for the decomposition of various components of plant material, i.e., cellulose and hemicelluloses [61,62]. *Odoribacter* and Clostridielesvadin BB60 is also identified in the healthy intestinal microbiota and is involved in the production of SCFAs (e.g., butyrate), which, play an important role in the maintenance of the epithelial barrier and intestinal homeostasis, and also have anti-inflammatory and immunomodulatory properties [63]. Moreover, butyrate a fermentation product of intestinal bacteria, has been reported to modulate brain functions by inhibiting histone deacetylase and act as antidepressant through the induction of the histone hyperacetylation in mice [64]. In turn *Alistipes* can cause both positive and negative effects, which is often associated with mood disorders, however, during the prebiotic diet, it may work beneficially by producing SCFAs [65]. It is worth noting that *Alistipes* species are indole-positive, which may affect the availability of tryptophan, which is the precursor of serotonin [66]. On the other hand, stress can activate kynurenine pathway enzymes, which may reduce the amount of tryptophan available for serotonin synthesis, making kynurenines important factors in the pathogenesis of anxiety-depressive disorders [67]. Perhaps the indirect role of these intestinal bacteria in maintaining normal levels of serotonin could have contributed to the alleviation of anxiety symptoms in EPM and FST tests in CUMS induce mice supplemented with INU.

Analysis of the 16S rRNA sequencing results for the FLU CUMS group identified 8 biomarker taxa: Enterobacteriaceae, *Escherichia-Shigella*, *Enterobacter*, Desulfovibrionaceae, *Lachnospiraceae ASF356*, *Anaerotruncus*, *Roseburia* and *Intestinimonas*. Moreover, the abundance of Proteobacteria was the highest in this group. Similar studies evaluating the effect of 3 weeks of FLU administration at a dose of 12 mg/kg on the gut microbiota in the CUMS mouse model were conducted by Sun et al. [68], where the Lefse analysis also showed an increased abundance of Proteobacteria. Since *Roseburia*, *Anaerotruncus*, *Intestinimonas* and *Lachnospiraceae ASF 356* (also identified in our research) belong to SCFAs producing commensal bacteria affecting immunity and anti-inflammatory effects, they can be related to health markers [69,70,71]. Additionally, SCFAs are able to pass from the intestines to the systemic circulation using passive diffusion of undissociated SCFAs or hydrogen- (MCT-1) and sodium-coupled (SMCT-1) transporters on epithelial cells [72]. In turn, Desulfovibrionaceae is known to affect the signaling of nerve cells and may contribute to the improvement of cognitive functioning, spatial learning and memory, and neuroprotection [73], as we could see in our MWM test results. In addition, we observed an increased abundance of the Enterobacteriaceae family (in particular, the *Enterobacter* and *Escherichia-Shigella* genus). It should be noted, that a large group of them is proved to be harmless and disease-free species living in the digestive tract of animals, whereas the other are pathogenic and may contribute to the development of intestinal disease [74]. The results obtained from our study indicated that FLU (12 mg/kg) administered for the last 3 weeks of CUMS induction alleviated stress-induced intestinal dysbiosis in CUMS mice, while when used in healthy animals, turned out to disrupt the symbiotic bacterial flora [26]. Such diverse results are certainly worth further preclinical studies.

Summing up the obtained results, TPB and INU turned out to have a potential as natural functional prebiotic supplements in the mouse model of CUMS. Additionally, we can assume that gut microbes and their fermentation products influence behavioral responses such as anxiety-like behavior, learning and memory processes, and hippocampal neurogenesis.

## 5. Conclusions

The presented study results for the first time showed that 10-week supplementation of TPB, a natural prebiotic, similarly to INU, may be beneficial in alleviating anxiety and depressive behaviors induced by chronic mild unpredictable stress in mice. Moreover, a long-term supplementation with TPB, like INU, turned out to have a beneficial effect on neurogenesis and cognitive functions in mice exposed to CUMS. Interestingly, the prebiotic diet alleviate stress-induced intestinal dysbiosis in all studied CUMS groups. Based on the obtained in vivo results from the moue CUMS model we can conclude that long-term supplementation with prebiotics such as TPB or INU may mitigate the negative effects of the everyday exposure to the unpredictable mild stress factors.

## Figures and Tables

**Table 1:** List of stress factors and the duration of the stimulus.

Days		CUMS
	Stress Factor	Duration of the Stimulus
1	Tilt the cage (45°)	4 h
2	Wet litter	24 h
3	Light at night	12 h
4	Restraint	2 h
5	Electric buzzer 90 dB	5 min
6	Water deprivation	24 h
7	Food deprivation	24 h

## Data Availability

The data supporting reported results can be found in the laboratory databases of Institute of Rural Health.

## Supplementary Materials

The following supporting information can be downloaded at: https://www.mdpi.com/article/10.3390/nu15092041/s1, Table S1: The complete composition of the intestinal microbiota.
